# A Single Low Dose of Dexmedetomidine Efficiently Attenuates Esketamine-Induced Overactive Behaviors and Neuronal Hyperactivities in Mice

**DOI:** 10.3389/fnhum.2021.735569

**Published:** 2021-10-12

**Authors:** Qinjun Chu, Kuicheng Zhu, Yafan Bai, Huijie Shang, Dongqing Zhang, Mingming Zhao, Ping Zheng, Xiaogao Jin

**Affiliations:** ^1^Department of Anesthesiology and Perioperative Medicine, Zhengzhou Center Hospital Affiliated to Zhengzhou University, Zhengzhou, China; ^2^Academy of Medical Sciences, Zhengzhou University, Zhengzhou, China; ^3^Department of Anesthesiology, Pain and Perioperative Medicine, The First Affiliated Hospital of Zhengzhou University, Zhengzhou, China; ^4^West Hoston Family Practice, Houston, TX, United States; ^5^Metabolic Disease Research Center, Zhengzhou Central Hospital Affiliated to Zhengzhou University, Zhengzhou, China; ^6^Center for Advanced Medicine, College of Medicine, Zhengzhou University, Zhengzhou, China; ^7^Research of Trauma Center, Zhengzhou Central Hospital Affiliated to Zhengzhou University, Zhengzhou, China

**Keywords:** esketmaine, dexmedetomidine, toxicity, behavior, anesthesia

## Abstract

**Introduction:** Esketamine (Esk) (S(+)-ketamine) is now used as an alternative to its racemic mixture, i. e., ketamine in anesthesia. Esk demonstrated more powerful potency and rapid recovery in anesthesia and less psychotomimetic side effects comparing with ketamine, but Esk could still induce psychological side effects in patients. This study was to investigate whether dexmedetomidine (Dex) can attenuate the Esk-induced neuronal hyperactivities in Kunming mice.

**Methods:** Dexmedetomidine 0.25, 0.5, and 1 mg/kg accompanied with Esk 50 mg/kg were administrated on Kunming mice to assess the anesthesia quality for 1 h. The indicators, such as time to action, duration of agitation, duration of ataxia, duration of loss pedal withdrawal reaction (PWR), duration of catalepsy, duration of righting reflex (RR) loss, duration of sedation, were recorded for 1 h after intraperitoneal administration. The c-Fos expression in the brain was detected by immunohistochemistry and Western Blot after 1 h of administration. Considering the length of recovery time for more than 1 h in Dex and Dex with Esk groups, other mice were repeatedly used to evaluate recovery time from the administration to emerge from anesthesia.

**Results:** Dexmedetomidine dose-dependently increased recovery time when administrated with Esk or alone. Dex combined with Esk efficiently attenuated the duration of agitation, ataxia, and catalepsy. Dex synergically improved the anesthesia of Esk by increasing the duration of sedation, loss of RR, and loss of PWR. Esk induced the high expression of c-Fos in the cerebral cortex, hippocampus, thalamus, amygdala, hypothalamus, and cerebellum 1 h after administration. Western Blot results indicated that Dex at doses of 0.25, 0.5, and 1 mg/kg could significantly alleviate the Esk-induced c-Fos expression in the mice brain.

**Conclusion:** Dexmedetomidine ranged from 0.25 to 1 mg/kg could improve the anesthesia quality and decreased the neuronal hyperactivities and the overactive behaviors when combined with Esk. However, Dex dose-dependently increased the recovery time from anesthesia. It demonstrated that a small dose of Dex 0.25 mg/kg could be sufficient to attenuate Esk-induced psychotomimetic side effects without extension of recovery time in Kunming mice.

## Introduction

Ketamine is a mixture of the two enantiomers, namely, esketamine (Esk) (S (+)-ketamine) and R (–)-ketamine, which can produce the so-called “dissociative” anesthesia state. The dissociative situation is characterized by strong analgesia and small hypnotic properties (Marland et al., 2013; Bozymski et al., 2020; Jelen et al., 2021). Patients may experience hallucination, catalepsy, amnesia, perception alteration, and out-of-body sense during ketamine anesthesia (Bokor and Anderson, 2014; Grabski et al., 2020). This special anesthesia state indicates that ketamine or Esk induced excitatory activity in some brain regions (Herrera and Robertson, 1990; Vaisanen et al., 2004; Tian et al., 2018). Olney et al. (1989) reported that N-methyl-D-aspartate (NMDA) antagonist could cause transient reversible vacuolation in neurons in the posterior cingulate cortex of rats (Olney et al., 1991; Nowacka and Borczyk, 2019). These neuronal vacuolations come from the lysis of mitochondria, which was injured during the overexcitation. The neurotoxicity induced by ketamine remains a great concern in clinical practice. Esk, the S (+)-ketamine, was introduced into clinical practice with more potency, less psychotic effects than ketamine and its right-hand enantiomer, R (–)-ketamine (Bauer et al., 2019).

Esketamine displays 2–3-fold more efficient than ketamine. Comparing with ketamine, Esk demonstrates faster and smoother recovery with less respiratory inhibition and less psychotomimetic side effects (Himmelseher and Pfenninger, 1998). However, it was reported that Esk could still induce dissociation disorder during the therapy of treatment-resistant depression (TRD) (Himmelseher and Pfenninger, 1998). Esk may be a breakthrough therapeutic in TRD in the last 20 years due to its rapid action (Kaur et al., 2021). The action occurs within 2 h after intranasal administration and produces a significant effect within 2 days; however, most first-line antidepressants take 4–6 weeks to attain their full effects (Kaur et al., 2021). The dissociation disorder of Esk during TRD included a distortion of time and space, illusions, derealization, and depersonalization (Bozymski et al., 2020). Esk and ketamine exert their anesthesia effects through antagonizing NMDA glutamate receptors. But it is reported that ketamine-induced psychopathology results from glutamate increase in the anterior cingulate cortex (Stone et al., 2012). The increased glutamate was thought to inhibit GABAergic interneurons by ketamine. Therefore, the drug that can activate GABA receptors can be used to reduce Esk-induced psychoactive side effects. These drugs included dexmedetomidine (Dex), clonidine, propofol, midazolam, and diazepam.

Dexmedetomidine and clonidine are agonists of alpha-2 adrenergic receptors in the brainstem. Activation of alpha-2 adrenergic receptors produces sedation by stimulating inhibitory neurons such as GABAergic interneurons (Stone et al., 2012). Stimulation of inhibitory neurons decreases sympathetic outflow of the central nervous system, resulting in sedation, blood pressure decrease, and heart rate decrease. Dex was widely used with ketamine or Esk to prevent tachycardia, hypertension, salivation, and emergence phenomena. However, it is still uncertain if Dex can prevent the psychotic effects and neuronal hyperactivities from Esk when used together (Calhoun et al., 2015). The purpose of this study was to determine whether Dex could efficiently prevent Esk-induced psychotic effects and neuronal hyperactivities in mice.

## Materials and Methods

### Ethics Statement

This study was approved by the Ethics Committee of Zhengzhou Central Hospital and was consistent with the Chinese Animal Welfare Act.

### Animals

Male Kunming mice aged 1 month, weighing around 25 g, were purchased from the Animal Center of the Medical College of Zhengzhou University. Mice were housed (6–9 mice per cage) in individually ventilated cages with wood bedding in accordance with the Chinese guidelines. Food and water were freely accessible to the mice. The room temperature was maintained at 22 ± 2°C and a humidity of 45–60%. Animals were maintained under a 12-h light-dark cycle. The mice were acclimated to circumstances 2 h before the interventions.

### Drugs

Esketamine was purchased from Hengrui Medicine Co., Ltd. (Lianyungang, Jiangsu Province, China). Dex was commercially available from Hengrui Pharmaceutical Co., Ltd (Jiangsu, Chinese medicine standard word H20090248, 2009-05-31, China).

### Experimental Design

Fifty-two mice were randomly assigned to eight groups (Table 1). All mice in the table were sacrificed under deep anesthesia 1 h after treatment to harvest brains for immunohistochemistry and Western Blot. The brains were longitudinally cut into two halves. One half was immersed in 4% of paraformaldehyde for immunohistochemistry, and another half was immediately frozen in −80°C for protein extraction for Western Blot. The mice were supplied with oxygen and kept warm during the experiment. For the mice with deep anesthesia, the tail oxygen saturation (SpO_2_) was monitored to keep it over 90% by adjusting oxygen flow. The mice in the saline group were intraperitoneally injected with the same volume of saline as the Esk and Dex. The mice in the Esk group were intraperitoneally administrated with 50 mg/kg Esk. The mice in Dex 0.25 mg/kg group were intraperitoneally administrated with 0.25 mg/kg Dex. The mice in Dex 0.50 mg/kg were intraperitoneally administrated with 0.5 mg/kg Dex. The mice in Dex 1.0 mg/kg were intraperitoneally administrated with 1 mg/kg Dex. In the Dex 0.25 mg/kg + Esk group, Dex 0.25 mg/kg and Esk 50 mg/kg were mixed in one syringe for intraperitoneal administration for each mouse. In the Dex 0.5 mg/kg + Esk 50 mg/kg group, Dex 0.5 mg/kg and Esk 50 mg/kg were mixed in one syringe for intraperitoneal administration for each mouse. In the Dex 1 mg/kg + Esk group, Dex 0.5 mg/kg and Esk 50 mg/kg were mixed in one syringe for intraperitoneal administration for each mouse.

**Table 1:** The mice groups treated in this study.

**Groups**	**Sample size**	**Treatment (ip)**
Saline	*n =* 6	Saline
Esk	*n =* 9	Esketamine 50 mg/kg
Dex 0.25 mg/kg	*n =* 6^*^	Dexmedetomidine 0.25 mg/kg
Dex 0.5 mg/kg	*n =* 6^*^	Dexmedetomidine 0.50 mg/kg
Dex 1.0 mg/kg	*n =* 6^*^	Dexmedetomidine 1.0 mg/kg
Dex 0.25 mg/kg+Esk	*n =* 6#	Dexmedetomidine 0.25 mg/kg+Esketamine 50 mg/kg
Dex 0.5 mg/kg+Esk	*n =* 6#	Dexmedetomidine 0.50 mg/kg+Esketamine 50 mg/kg
Dex 1 mg/kg+Esk	*n =* 7#	Dexmedetomidine 1.0 mg/kg+Esketamine 50 mg/kg

* *Another six mice in Dex groups were repeatedly used three times to evaluate the recovery time from low dose of dexmedetomidine to high dose allowing washout for 1 week between the experiments. #Another six mice were also assigned into Dex+Esk groups to assess the recovery time allowing repeated use*.

The Esk 50 mg/kg through intraperitoneal administration was referred to Bauer et al. (2019). The Dex dose was referred to Burnside et al. (2013). Burnside et al. used dexmedetomidine by a dose of 0.5 mg/kg. We used a higher dose of 1 mg/kg, a middle dose of 0.5 mg/kg, and a lower dose of 0.25 mg/kg to constitute a dose-effect curve in this study.

### Experimental Procedure

Each mouse was placed alone in one cage for observation after intraperitoneal administration of the drugs. The mice were continually observed for 1 h before sacrifice. The animal behaviors, such as ataxia, catalepsy, excitation, sedation, pedal withdrawal reaction (PWR), and RR, were continuously observed for 1 h. In this study, we found that the effects of Esk lasted about 30 min, the effects of Dex lasted over 60 min, and there was almost no overactive behavior after the combination of Esk and Dex except transient ataxia. Therefore, we observed for 1 h to cover the effects of both Esk and Dex.

Time to action was defined as the time from the intraperitoneal injection to the appearance of any signs of ataxia, excitation, sedation, and catalepsy. Ataxia was characterized by the loss of coordination and balance in walking and keeping posture. Duration of ataxia was regarded as the accumulative time for ataxia during monitoring (Cendelin, 2014).

Excitation was defined as a state of excitement including myoclonic twitching and aimless running. Duration of excitation was the accumulative time of excitation during the 1-h observation (Bauer et al., 2019).

Catalepsy was defined as a state of immobilization combined with a noticeably increased muscle tone. Duration of catalepsy was the accumulative time of catalepsy during the monitoring (Bauer et al., 2019).

Sedation was defined as a state of immobilization, sleep-like posture, but still responsive to stimulation. Duration of sedation was recorded as the accumulative time of the situation of sedation (Taiji, 2004; Calhoun et al., 2015).

Pedal withdrawal reaction was used to evaluate the effects of analgesia after administration of Esk or Dex. The positive reaction was recorded when mice attempt to withdraw the limb to the toe pinch, and the negative reaction was recorded when mice had no response to the toe pinch. The pinch should not cause any injuries to the toes. Loss of PWR start was defined as three successive negative PWR. Recovery of PWR was defined as three positive PWR. Duration of loss of PWR is defined as the accumulative time of loss of PWR (Bauer et al., 2019).

Righting reflex was used to assess the anesthesia effect after administration of Esk or Dex (Burnside et al., 2013). The mouse was physically rolled into lateral recumbency by hand to evaluate RR. Loss of RR was recorded when the mouse was unable to recover from lateral recumbency to ventral recumbency without assisting three times. Recovery of RR was recorded when the mouse was able to roll unassistedly from lateral to sternal recumbency three times. Duration of loss of RR was defined as the accumulative time of loss of RR.

Recovery time was defined as the time from the injection of anesthetics to regaining of RR, PWR, and maintenance of a ventral recumbence posture (Bauer et al., 2019). Since recovery time is longer than 1 h, another 13 mice were used to observe beyond more than 1 h until complete recovery without sample collection. After 1-h observation, mice were terminally anesthetized by chloral hydrate and euthanized by decapitation, and the brain tissues were retrieved for immunohistochemistry and Western Blot.

### Immunohistochemistry

Mice brains were put into 4% of paraformaldehyde for at least 24 h at 4°C. The brains were embedded in paraffin after dehydration and clearance. The paraffin-embedded tissue blocks were cut into a section at 4 μm thickness on a microtome. Sections on glass slides were deparaffinized and rehydrated before immunohistochemistry. Sections were boiled in citrate buffer (pH 6.0) for 20 min for antigen retrieval. The endogenous peroxidase was blocked by incubating in 3% of H_2_O_2_ solution for 10 min at room temperature. The primary antibody c-Fos (Bioss, China) was added onto the sections for incubating overnight at 4°C. The biotinylated secondary antibody and streptavidin-HRP were sequentially incubated with the sections. The antibody was stained using 3,3′-diaminobenzidine (DAB) substrate solution (Zsbio, China). The slides were counterstained by hematoxylin. The slides were dehydrated and cleared before mounting coverslips. Sections were visualized under a microscope (Olympus, Japan) 100 and 400 times.

### Western Blot

Protein was extracted using radioimmunoprecipitation assay (RIPA) buffer (Epizyme, China) containing cocktail proteinase inhibitors. The protein concentration was quantified with a bicinchoninic acid (BCA) protein assay kit (Epizyme, China). The same amount of protein was run on sodium dodecyl sulfate (SDS)-polyacrylamide gels in the Tris/SDS buffer system and then transferred into polyvinylidene difluoride (PVDF) membranes. The membranes and the primary antibodies c-Fos (Bioss, China) were incubated together overnight and then incubated with horseradish peroxidase (HRP)-conjugated secondary antibody (Abbkine, China). The glyceraldehyde-3-phosphate dehydrogenase (GAPDH) antibody (Boster, China) was used as an internal reference antibody. The protein expression was analyzed using an electronic shelf label (ESL) scanner with Omni-ECL Enhanced Pico Light Chemiluminescence Kit (Epizyme Biotech, China), and signal intensities of the protein expression were quantified using NIH Image/J software (National Institutes of Health, Bethesda, MD).

### Statistical Analysis

In this study, behavioral data presented are described as median with 25–75 percentiles (median, lower quartile-upper quartile). Median analysis was performed using the Kruskal-Wallis rank-sum test followed by the *post-hoc* test using R software (R Foundation for Statistical Computing, Vienna, Austria). The *P*-value is adjusted by the false discovery rate (FDR) procedure of Benjamini-Hochberg. The expression of Cellular protooncogene Fos (c-Fos) in Immunohistochemistry (IHC) was evaluated by calculating the numbers of staining positive cells in a high-power field (400 times). The IHC results were presented by mean ± SD (M±SD) and analyzed by the independent *t*-test to just compare the saline and Esk groups. Western Blot results were presented as mean ± SD (M±SD). The ANOVA test was used to analyze the c-Fos expression differences in all groups. *P* < 0.05 was considered as the significant difference.

## Results

### General Behavioral Performance After Intraperitoneal Administration of Esk and Dex

After injection of Esk, the mice appeared gradually ataxia including the loss of coordination and balance in walking and keeping posture. Subsequently, the mice displayed excitation, which is characterized by myoclonic twitching and aimless running. During the excitation, some mice showed catalepsy, which demonstrated immobilization associated with intensive muscle tone all over the body, something like arched-back rigidity. After excitation and catalepsy, the mice returned to ataxia again and regained coordination and balance gradually. However, after intraperitoneal injection of Esk with Dex, mice demonstrated less ataxia and excitation without any catalepsy. One mouse died in the Dex 1 mg/kg + Esk group due to respiratory depression.

### Time to Action

**Figure 1:**
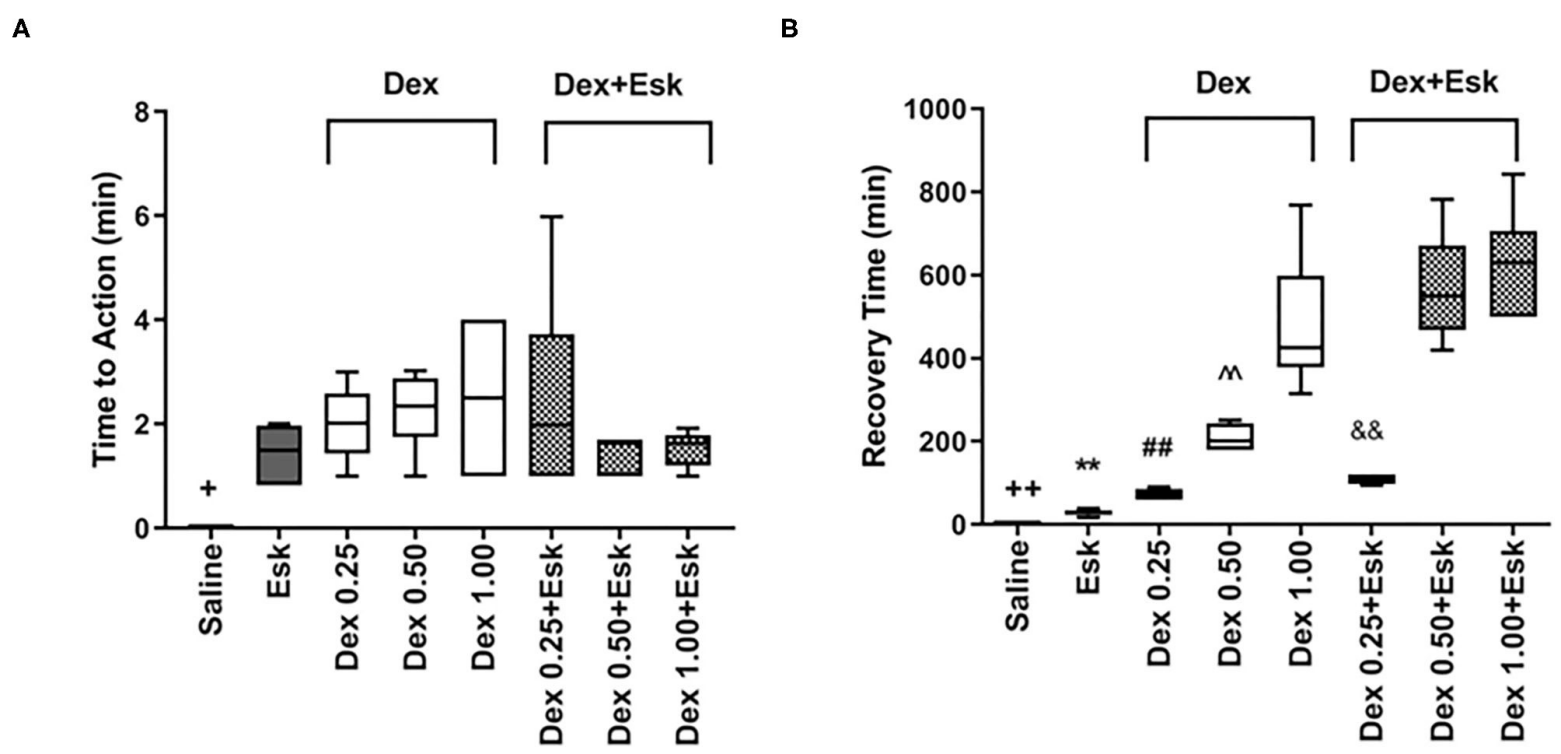
Time to action and time to recovery after injection. **(A)** ^+^*P* < 0.05, compared with esketamine (Esk), dexmedetomidine (Dex), and Dex + Esk groups. **(B)** Recovery time. ^++^*P* < 0.01, compared with Esk, Dex, and Dex + Esk group. **P < 0.01, compared with Dex and Dex + Esk group. ^##^*P* < 0.01, compared with Dex 0.5 mg/k, Dex 1 mg/kg, and Dex 0.25 mg/kg + ESk. ^∧∧^*P* < 0.01, compared with Dex 1 mg/kg and Dex + Esk. ^&&^*P* < 0.01, compared with Dex 0.5 mg/kg + Esk and Dex 1 mg/kg.

All groups showed similar onset times. There was no significant difference in onset time between these groups (Figure 1A).

### Recovery Time

Recovery time was from 17.25 to 39 min with a median of 30 min in Esk groups. In combination with Esk, Dex dose-dependently extended recovery time. There was no difference between Dex + Esk groups and corresponding Dex groups (Figure 1B).

### Overactive Behaviors

**Figure 2:**
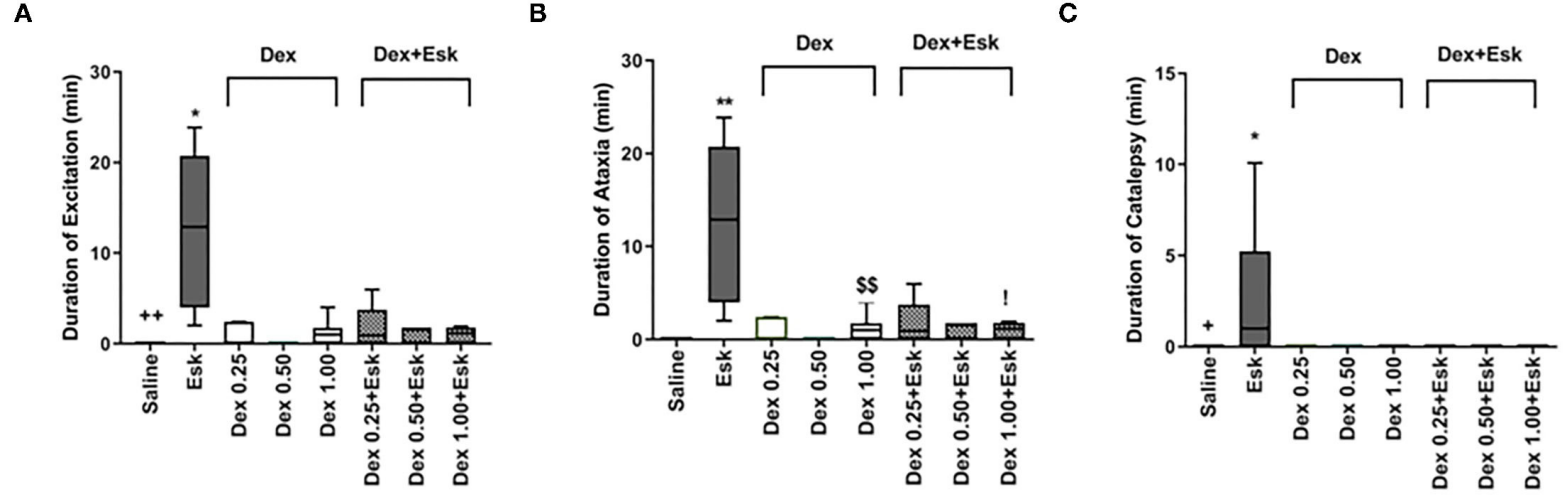
Overactive behaviors including duration of excitation, ataxia, and catalepsy during 1 h observation after injection. **(A)** Duration of excitation. **P < 0.01, compared with saline, Dex, and Dex + Esk groups. **(B)** Duration of ataxia. **P < 0.01, compared with saline, Dex, and Dex + Esk groups. ^$$^*P* < 0.01, compared with saline, ^!^*P* < 0.05, compared with Saline. **(C)** Duration of catalepsy. **P < 0.01, compared with saline, Dex, and Dex + Esk groups.

The Esk-alone group showed a longer duration of ataxia, excitation, and catalepsy than other groups (Figures 2A–C). There was no difference between the rest groups in duration of ataxia and excitation (Figures 2A,B). Catalepsy was a unique phenomenon for Esk. The other groups did not display any sign of catalepsy (Figure 2C).

### Anesthesia Assessment

**Figure 3:**
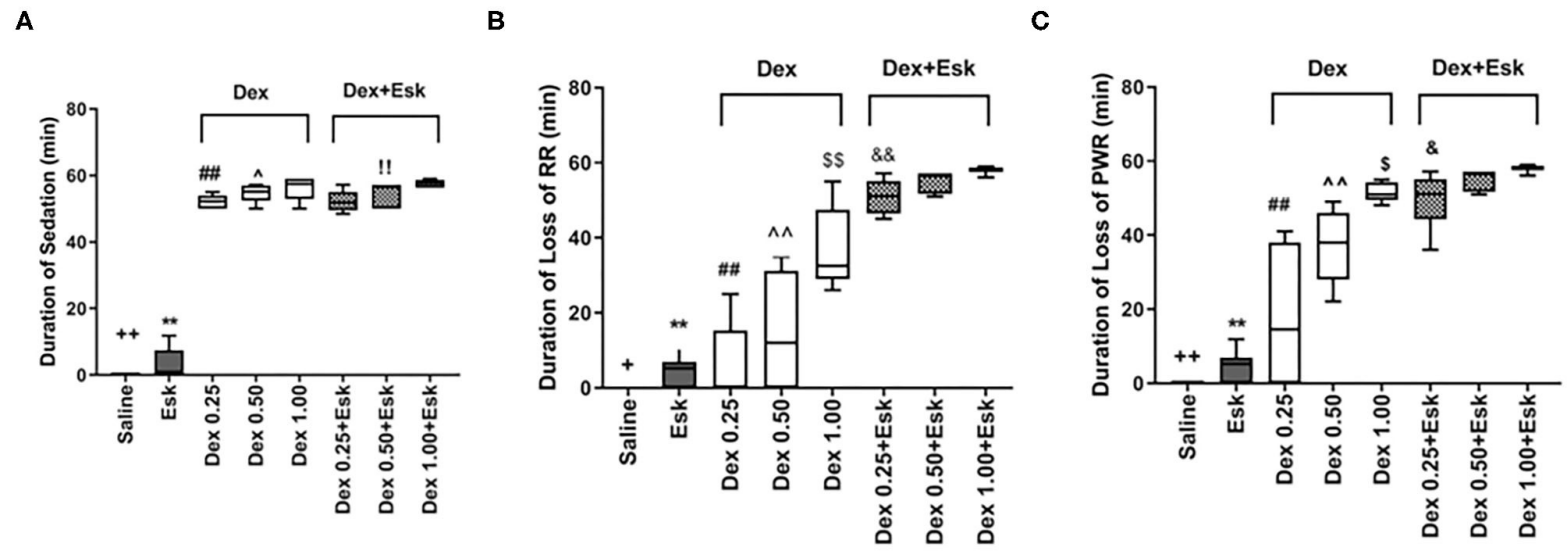
Assessment of anesthesia qualities including duration of sedation, loss of righting reflex (RR), and loss of paw withdraw reaction during 1 h observation. **(A)** Duration of sedation. ^++^*P* < 0.01, compared with Esk, Dex, and Dex + Esk. **P < 0.01, compared with Dex and Dex + Esk groups. **(B)** Duration of loss of RR. ^+^*P* < 0.05, compared with Esk.**P < 0.01, compared with Dex 1 mg/kg and Dex + Esk groups. ^##^*P* < 0.01, compared with Dex 1 mg/kg and Dex + Esk. ^∧∧^*P* < 0.01, compared with Dex 0.5 + Esk. ^&&^*P* < 0.01, compared with Dex 0.25 mg/kg + Esk. ^$$^*P* < 0.01, compared with Dex 1 mg/kg + Esk. ^&&^*P* < 0.01, compared with Dex 1 mg/kg + Esk. **(C)** Duration of loss of pedal withdrawal reaction (PWR). ^++^*P* < 0.01, compared with Esk, Dex, and Dex + Esk groups. **P < 0.01, compared with Dex and Dex + Esk groups. ^##^*P* < 0.01, compared with Dex 0.5 mg/kg, Dex 1 g/kg, and Dex + Esk groups. ^∧∧^*P* < 0.01, compared with Dex 1 mg/kg and Dex + Esk groups. ^$^*P* < 0.05, compared with Dex 1 mg/kg + Esk. ^$^*P* < 0.05, compared with Dex 1 mg/kg + Esk.

The Esk group displayed the shortest duration of sedation among all groups. There was no significant difference in duration of sedation between the other groups, but the duration of sedation was only observed within 1 h (Figure 3A). There was the longest duration of loss of RR and PWR in the Esk group with 1 mg/kg Dex (Figures 3B,C). Esk 0.5 and 0.25 mg/kg Dex had a similar duration of loss of PWR and RR. Esk alone shows the shortest duration of loss of PWR and RR among all groups. Dex with Esk had a longer duration of loss of PWR and RR than Dex alone. The Dex 1 mg/kg group had a longer duration of loss of PWR and RR than 0.5 mg/kg and 0.25 mg/kg Dex. There was no difference between 0.5 and 0.25 mg/kg Dex groups in the duration of loss of PWR and RR (Figures 3B,C).

### c-Fos Expression in Mice Brain

**Figure 4:**
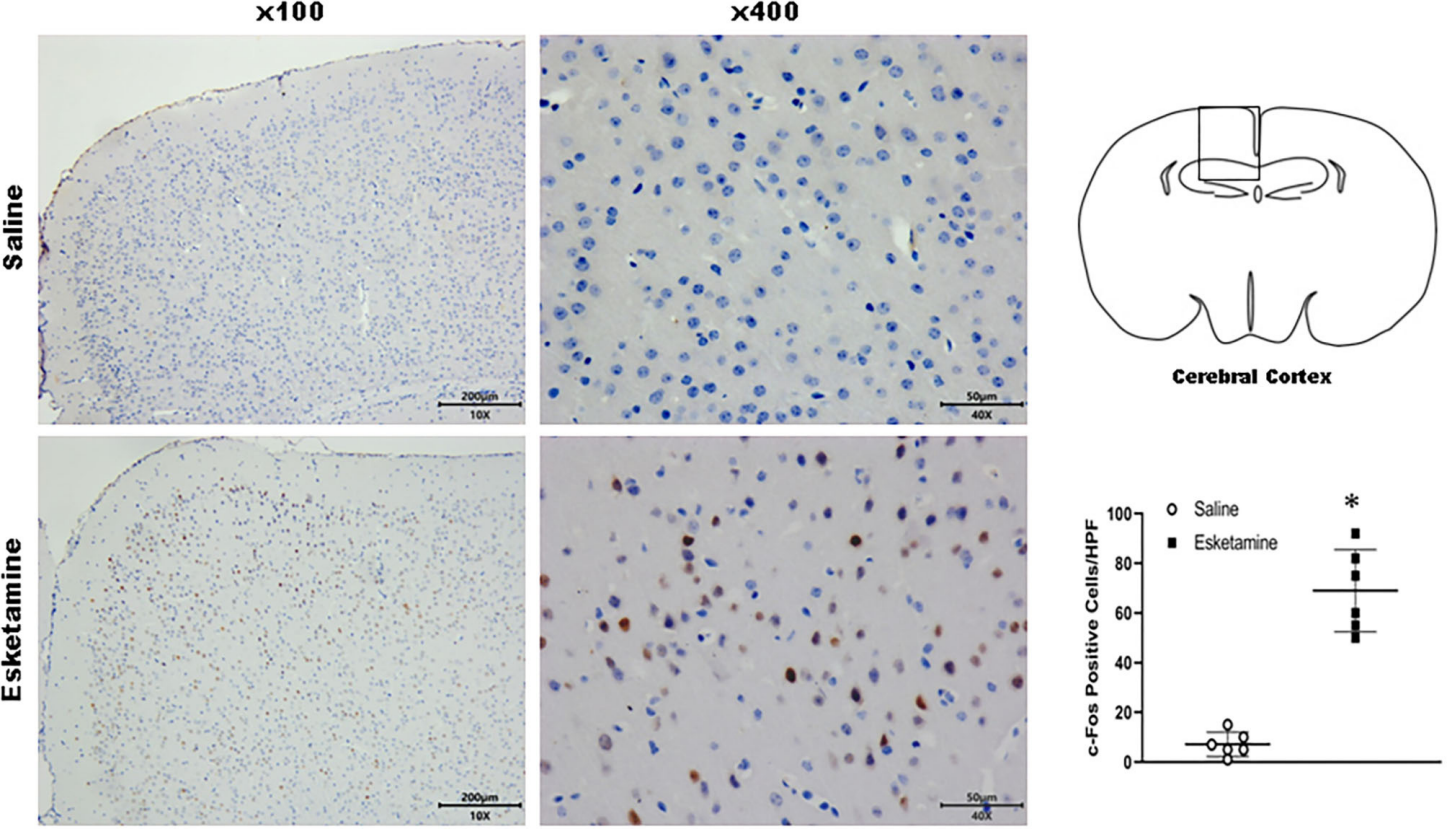
Expression of c-Fos in the cerebral cortex in the mice brain regions 1 h after injection of Esk. *P < 0.01, compared with the saline group (*n* = 6).

**Figure 5:**
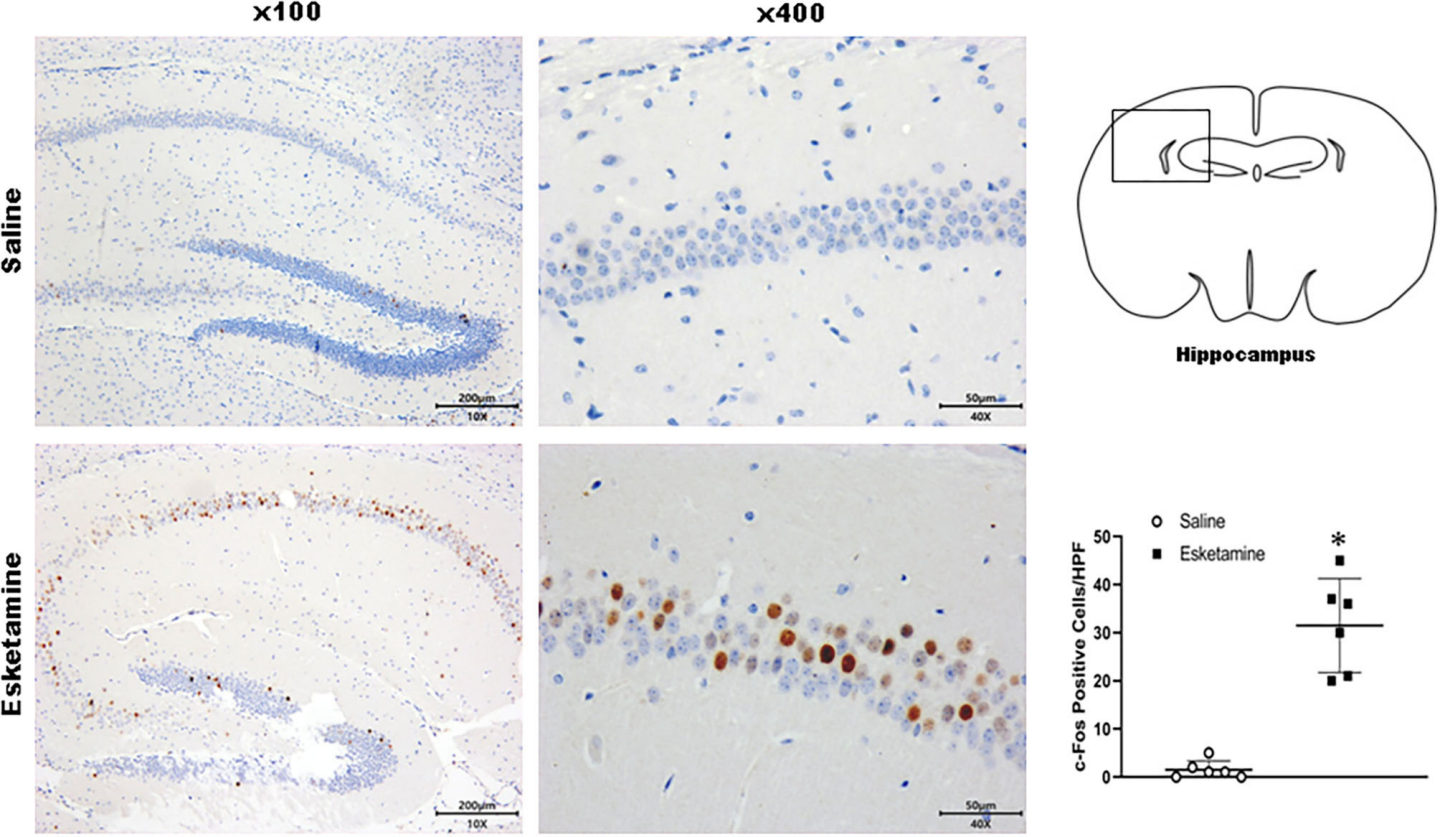
Expression of c-Fos in the hippocampus in the mice brain regions 1 h after injection of Esk. *P < 0.01, compared with the saline group (*n* = 6).

**Figure 6:**
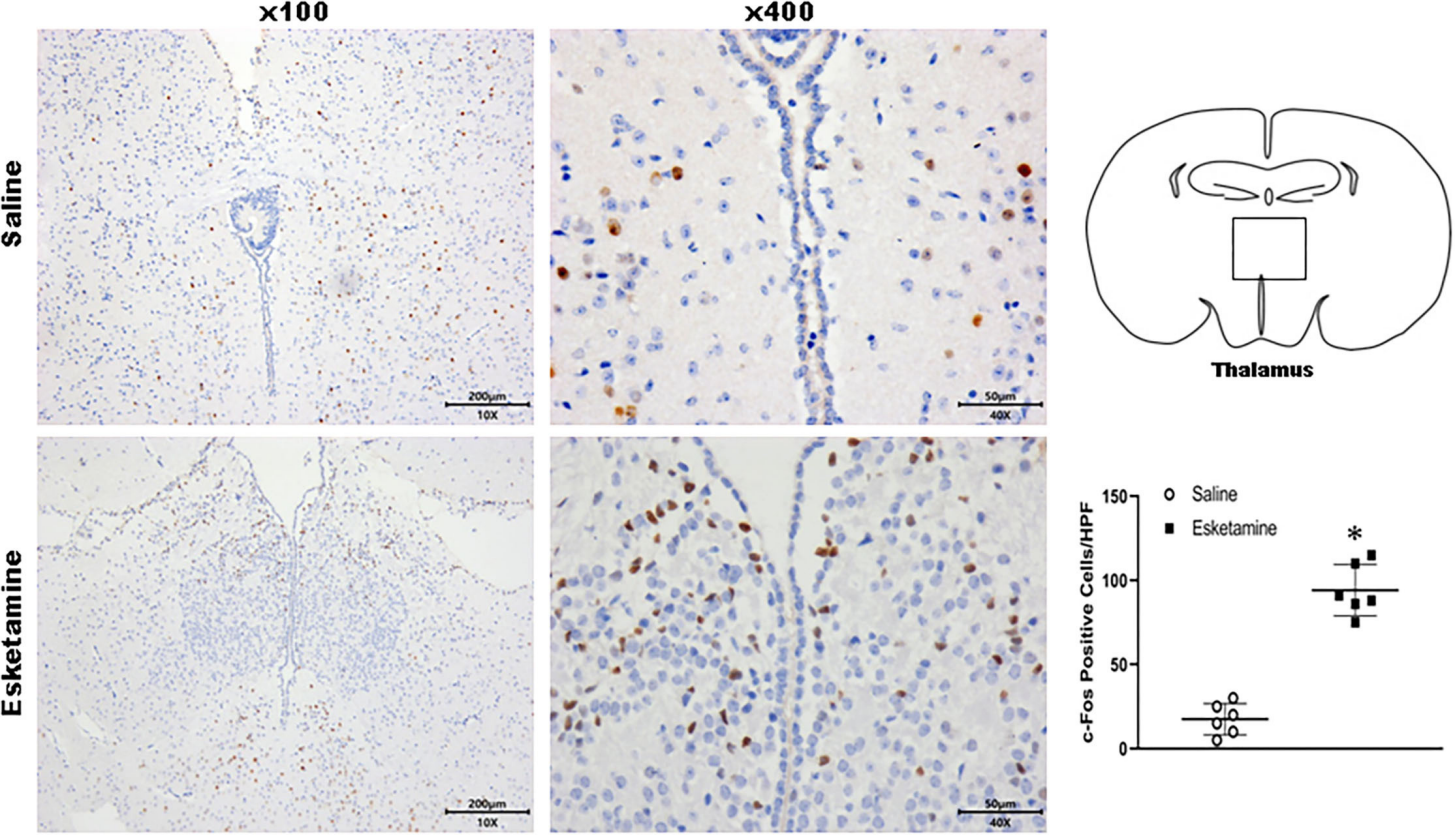
Expression of c-Fos in the thalamus in the mice brain regions 1 h after injection of Esk. *P < 0.01, compared with the saline group (*n* = 6).

**Figure 7:**
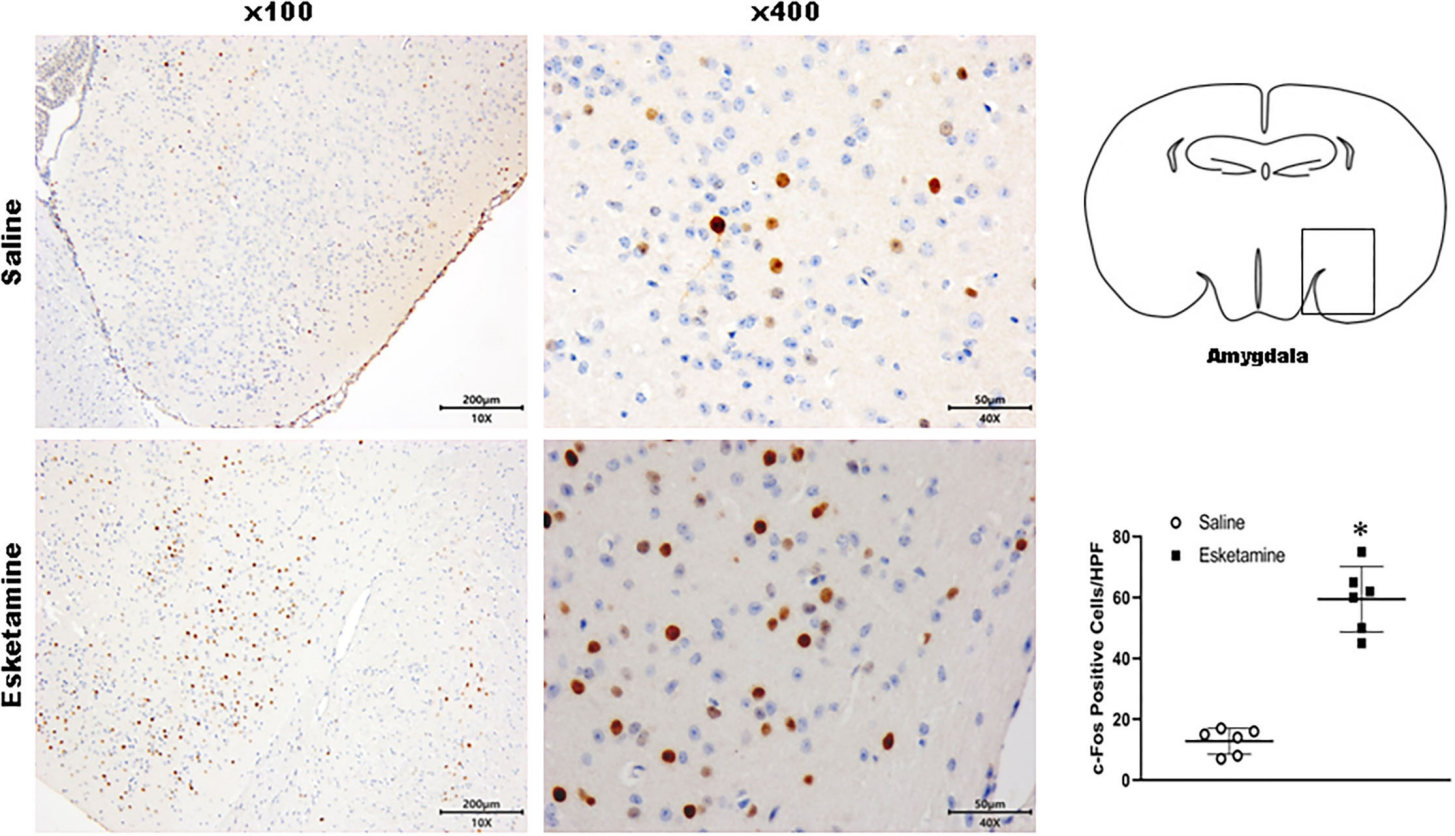
Expression of c-Fos in the amygdala in the mice brain regions 1 h after injection of Esk. *P < 0.01, compared with the saline group (*n* = 6).

**Figure 8:**
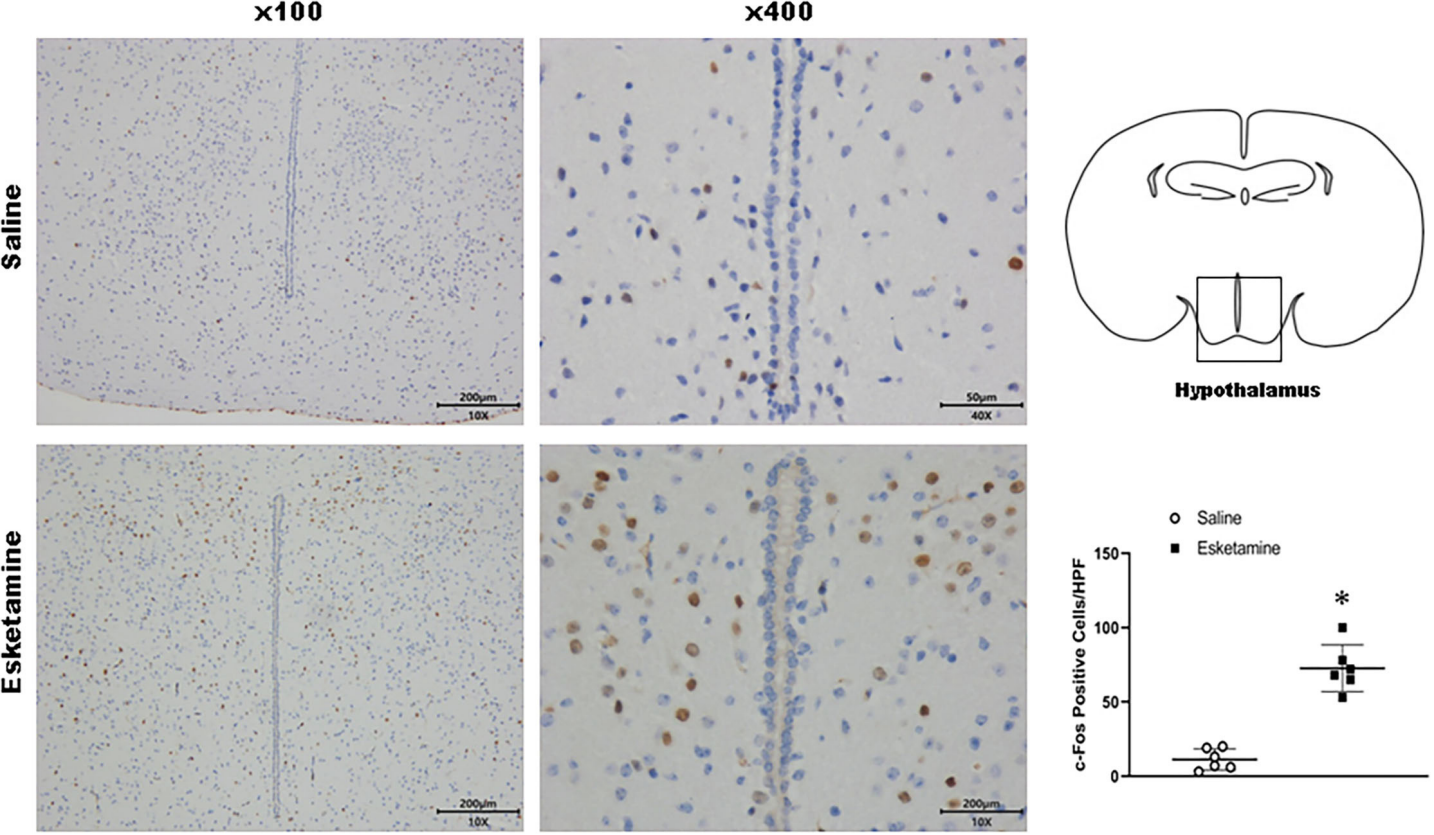
Expression of c-Fos in the hypothalamus in the mice brain regions 1 h after injection of Esk. *P < 0.01, compared with the saline group (*n* = 6).

**Figure 9:**
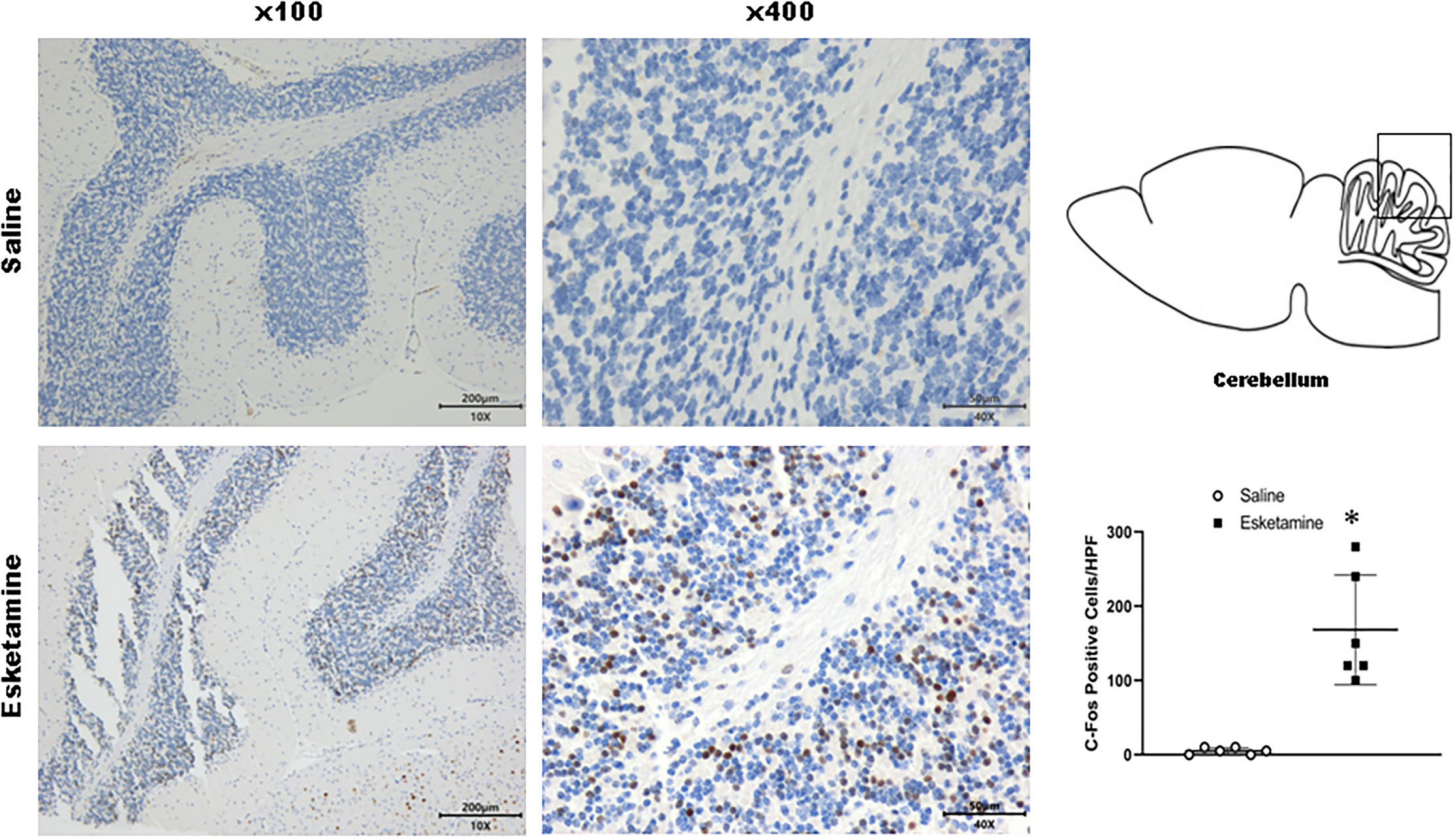
Expression of c-Fos in the cerebellum in the mice brain regions 1 h after injection of Esk. *P < 0.01, compared with the saline group (*n* = 6).

c-Fos protein induced by Esk increased in neurons in the cerebral cortex, hippocampus, thalamus, amygdala, hypothalamus, and cerebellum in mice brains (Figures 4–9 and Supplementary Figure 1). Dex 0.25, 0.5, and 1 mg/kg decreased significantly the level of c-Fos expression in these regions triggered by Esk, which was presented in Western Blot results. Figures 4–9 show the representative results related to Esk -induced neuronal hyperactivities.

### Western Blot Result

**Figure 10:**
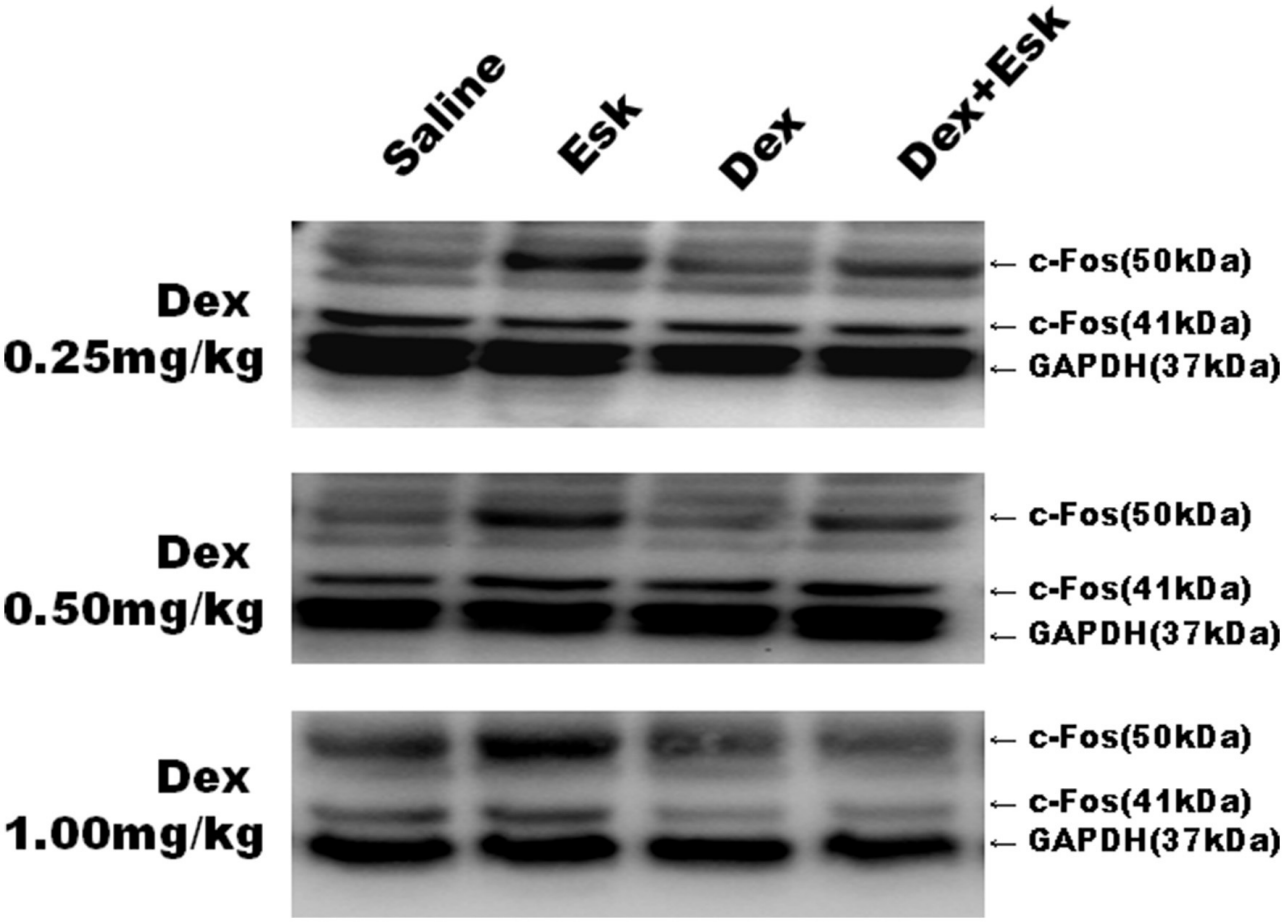
Representative Western Blot results for c-Fos expression in mice whole brain at 1 h after injection.

**Figure 11:**
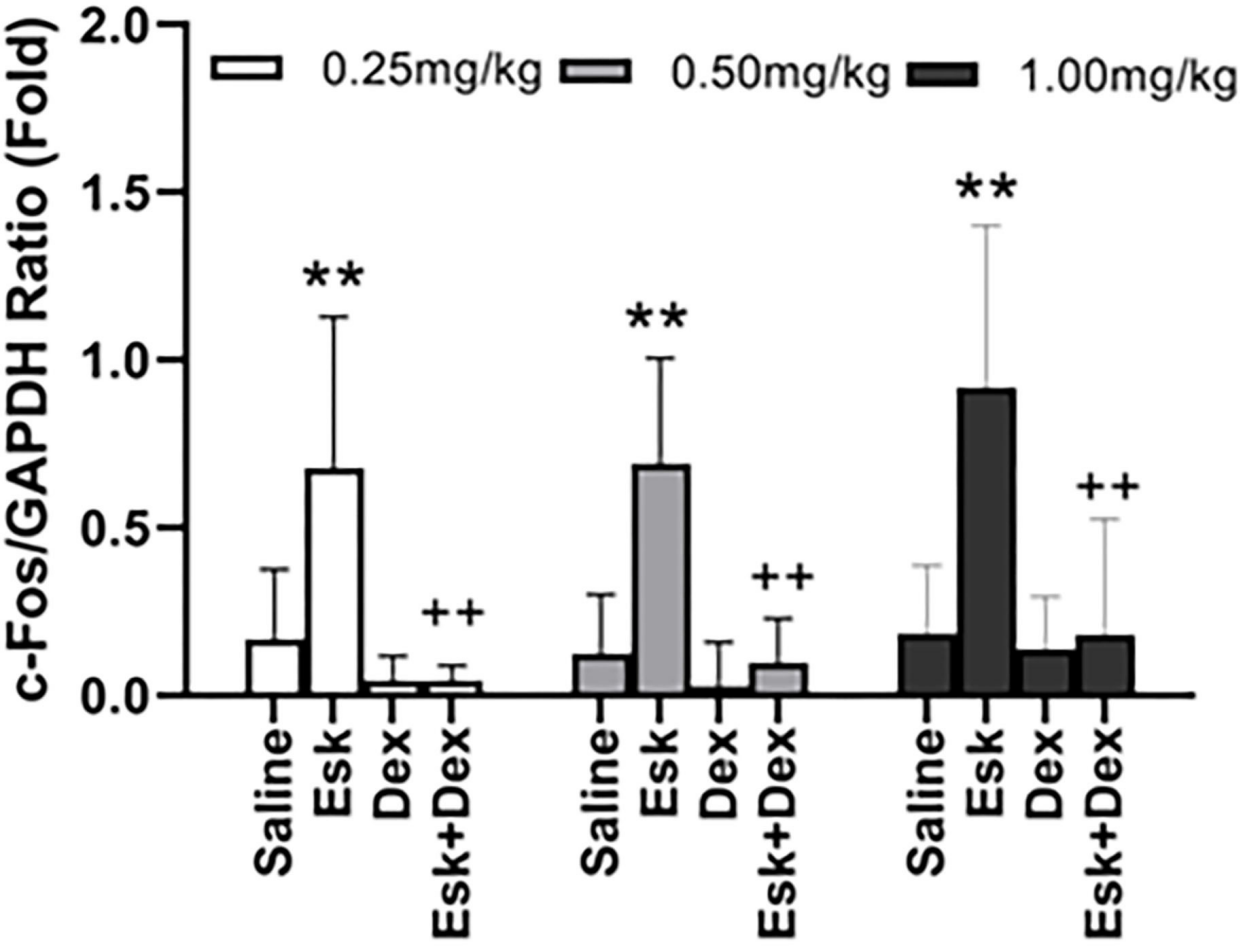
Group analysis of c-Fos expression over the glyceraldehyde-3-phosphate dehydrogenase (GAPDH) ratio in mice whole brain at 1 h after injection. **P < 0.01, compared with saline and Dex groups. ^++^*P* < 0.01, compared with the Esk group.

Esketamine groups had higher levels of c-Fos than saline groups. Dex 0.25, 0.5, and 1 mg/kg with Esk showed less c-Fos expression than Esk alone. There was no significant difference in c-fos expression in Dex + Esk groups (*P* < 0.05) (Figures 10, 11).

## Discussion

Psychogenic effects were the major concern when ketamine or Esk was used in anesthesia. Dex, a highly selective α_2_ adrenoceptor agonist, was recommended to prevent ketamine-induced psychological effects and improve anesthesia quality (Calhoun et al., 2015). There is minimal knowledge regarding the efficacy of Dex on psychogenic effects of Esk. It is necessary to reveal the effect of Dex on Esk-induced psychological effects to guide clinical practice (Lee, 2019).

In this study, we found that the Esk-induced neuronal side effects were manifested as overactivities in mice behaviors and highly expressed c-Fos in mice brains; Dex at doses of 0.25, 0.5, and 1 mg/kg efficiently relieved Esk-induced neural overactivities including ataxia, excitation, and catalepsy; Dex at doses of 0.25, 0.5, and 1 mg/kg efficiently alleviated the Esk-induced neurotoxicity, which was manifested as the c-Fos expression in the mice brain; Dex and Esk synergically improved the anesthesia quality, which was assessed by PWR and RR; and Dex dose-dependently extended the recovery time, whether using it alone or in combination with Esk.

Ketamine and its enantiomer S(+)-ketamine isoform, Esk, both have dissociative effects (Zanos et al., 2018). The dissociative effects include loss of self, inability to move the body, and isolation of mind from body. The symptoms are similar to schizophrenia and are called schizophrenia-like changes (Marland et al., 2013). The patient with ketamine anesthesia will experience emergence phenomena, which manifested by floating sensation, vivid pleasant dreams, nightmares, hallucination, and delirium (Bokor and Anderson, 2014). It was revealed that Esk could induce neuronal hyperactivities in mice, which were manifested as high expression of c-Fos in this study. The neurotoxicity may relate to the behavioral changes after the administration of ketamine (Vaisanen et al., 2004). The abnormal behaviors including ataxia, excitation, and catalepsy were consistent with the c-Fos expression in brain regions, which contained the cerebral cortex, hippocampus, thalamus, hypothalamus, and cerebellum. c-Fos expression is widely used to indicate neuronal hyperactivities, especially high glutamatergic input and subsequent activation of NMDA receptors. Therefore, in this study, c-Fos was applied to confirm neuronal excitation (Cano-Ramirez et al., 2021). To prevent these side effects, midazolam, propofol, and Dex were widely used with ketamine or Esk. Dex was commonly used with Esk in animal anesthesia (Bauer et al., 2019). Because of using Dex antagonist, and atipamezole, Dex dose is usually high in animal anesthesia. No matter how much dose of Dex was used, atipamezole can reverse its effect in about 5 min (Bauer et al., 2019). However, there is no Dex antagonist for human so far. Therefore, it is important to determine the optimal dosage of Dex to prevent Esk-induced neurotoxicity in clinical practice.

In this study, we found that Dex 0.25, 0.5, and 1 mg/kg efficiently alleviated the Esk-induced overactive behaviors and neuronal toxicity. There was no difference between these doses in abnormal behaviors and c-Fos expression. But the recovery time was extended to about 10 h in the mice treated with the combination of 0.5 or 1 mg/kg Dex with Esk. The recovery time of the combination of 0.25 mg/kg Dex with Esk was about only 1.5 h. Considering recovery time, 0.25 mg/kg Dex is the priority over 0.5 or 1 mg/kg Dex to efficiently alleviate Esk-induced neuronal side effects in Kunming mice. In addition, in this study, the duration of anesthesia, which was demonstrated by loss of RR and PWR, was also about 1.5 h and fit for most minor surgery.

Dexmedetomidine was recommended to administrate intravenously by a loading dose of 1 μg/kg followed by an infusion of 0.2–0.7 μg/kg/h (Weerink et al., 2017; Reel and Maani, 2021). According to our results, first, it may be not necessary to infuse Dex continuously after a loading dose when using with Esk because Dex has a long half time (about 120 min in human) (Weerink et al., 2017); second, the loading dose should be decreased to improve side effects when using with Esk.

According to pharmacological mechanisms, Esk and Dex have opposite effects on the cardiovascular system. During anesthesia, Esk and ketamine have side effects of increased blood pressure and heart rate; however, Dex and clonidine lowered blood pressure and heart rate (Mondardini et al., 2019; Wang et al., 2019, 2021; Reel and Maani, 2021). Therefore, it is reasonable to combine Esk and Dex or clonidine in anesthesia, especially in pediatric anesthesia. It was reported that the combination of ketamine and clonidine was more effective than the nebulization of ketamine alone and caused no serious adverse effects including hemodynamic instability (Shekhar et al., 2019). Jigar Patel et al. also found that combined low-dose clonidine and ketamine-induced perioperative sedation and effective suppression of sympathetic response with stable hemodynamics (Patel et al., 2016). This study also showed that the combination of Dex and Esk could synergically enhance each other in sedation and analgesia. In this study, it was indicated that the duration of loss of RR, duration of loss of PWR, and duration of sedation in combination groups were longer than that in groups with corresponding doses of Dex or Esk alone. Therefore, combination administration extremely decreases the dose of Dex and Esk to get the desired anesthesia situation, which needs a higher dose when using alone. Less doses of Dex and Esk will produce less side effects. The side effects of Dex included hypotension, bradycardia, and respiratory depression, and extension of recovery time (Bokor and Anderson, 2014). This study focused on the recovery time, without assessment of hypotension, bradycardia, and hypothermia. Evidence has proved that less Dex causes less incidence of hypotension, bradycardia, and hypothermia (Burnside et al., 2013). In this study, one mouse with Dex 1.0 mg + Esk died due to respiratory depression during the experiment. Moreover, it was also indicated that a high dose of Esk or ketamine produces a high incidence of psychological effects in patients (Fedgchin et al., 2019; Popova et al., 2019; Bozymski et al., 2020). So, the combination of Dex and Esk not only improves the quality of anesthesia but also decreases their dose and the incidence of side effects of each other.

Respiratory depression is a severe side effect for both Esk and Dex. Both Esk and Dex must be used under monitoring and available breath support. However, it was widely accepted that Dex and clonidine have less effect on respiratory function due to their unique mechanism, alpha-2 agonist (Nguyen et al., 2017). However, in this study, we found that Dex can dose-dependently extend the recovery time. It implied that an unnecessary high dose of Dex may still cause respiratory depression in anesthesia. In fact, in this study, one mouse administrated with Dex and Esk died due to respiratory depression. So, attention should be paid to breathing depression when a high dose of Dex is used with any other anesthetics.

In this study, the mice were about 1 month old which is considered immature. This is the limitation of this study. The adult mice are about from 3 to 6 months. So, in this study, the interpretation of the findings may favor the administration of Esk and Dex in pediatric anesthesia. Another limitation of this study is that the number of mice in each group was small. Most subgroups had six mice. The small number of mice might not represent the general changes of behaviors for all mice.

## Conclusion

This study suggested that Dex at doses of 0.25, 0.5, and 1 mg/kg could efficiently alleviate Esk-induced neuronal side effects in mice. However, only 0.25 mg/kg Dex could efficiently prevent Esk-induced neuronal side effects without extension of recovery time. Therefore, consideration of recovery time, a single low dose of Dex could be sufficient to prevent the psychological effects induced by Esk in mice. These findings may help to guide the administration of Dex with Esk in clinical anesthesia to avoid the side effects of each other. A further clinical study is necessary to determine the optimal dosage of Dex to prevent Esk-induced neuronal side effects.

## Data Availability Statement

The original contributions presented in the study are included in the article/Supplementary Material, further inquiries can be directed to the corresponding author/s.

## Ethics Statement

The animal study was reviewed and approved by the Ethics Committee of Zhengzhou Central Hospital.

## Author Contributions

QC prepared and wrote the manuscript, performed data analysis, and contributed to manuscript review and preparation. KZ aided in study design, collected and analyzed data, and contributed to manuscript writing and review. YB collected data, prepared tables and figures, and contributed to manuscript review and preparation. HS prepared and performed the animal experiment. DZ contributed to the experiment of Western Blot. MZ prepared and performed an IHC experiment. PZ improved English writing. XJ designed this study, performed data analysis, contributed to manuscript writing and review, and supervised all aspects of this study. All authors read and approved the final manuscript.

## Conflict of Interest

The authors declare that the research was conducted in the absence of any commercial or financial relationships that could be construed as a potential conflict of interest.

## Publisher's Note

All claims expressed in this article are solely those of the authors and do not necessarily represent those of their affiliated organizations, or those of the publisher, the editors and the reviewers. Any product that may be evaluated in this article, or claim that may be made by its manufacturer, is not guaranteed or endorsed by the publisher.

## Supplementary Material

Supplementary Figure 1 The regions of c-Fos expression in the mice brain.
