# Supplementary figures and images for: A Single Low Dose of Dexmedetomidine Efficiently Attenuates Esketamine-Induced Overactive Behaviors and Neuronal Hyperactivities in Mice
Source: Front Hum Neurosci. 2021 Oct 12;15:735569. doi: 10.3389/fnhum.2021.735569 (PMC8545873; doi:10.3389/fnhum.2021.735569)

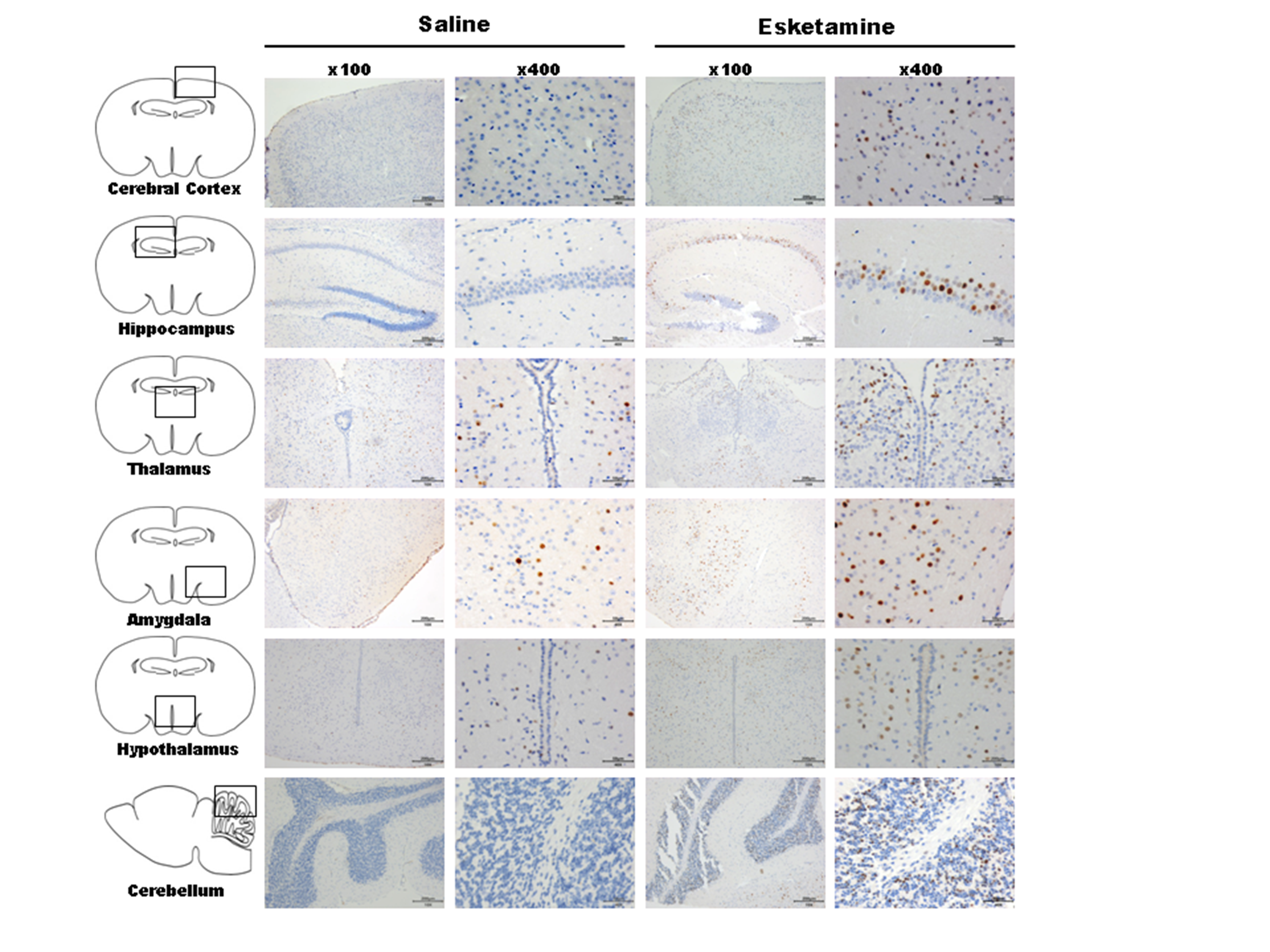

Supplement: Supplementary Figure 1 — The regions of c-Fos expression in the mice brain. [file Image_1.tif]
